# Dirhamnolipid Produced by the Pathogenic Fungus *Colletotrichum gloeosporioides* BWH-1 and Its Herbicidal Activity

**DOI:** 10.3390/molecules24162969

**Published:** 2019-08-16

**Authors:** Zhaolin Xu, Mengying Shi, Yongqing Tian, Pengfei Zhao, Yifang Niu, Meide Liao

**Affiliations:** Key Lab of Natural Pesticides & Chemical Biology, Ministry of Education, Department of Pesticide Science, South China Agricultural University, Guangzhou, Guangdong 510642, China

**Keywords:** biopesticide, bioherbicide, phytotoxin, rhamnolipid, biosurfactant, *Colletotrichum*, secondary metabolite, synergy

## Abstract

Fungal phytotoxins used as ecofriendly bioherbicides are becoming efficient alternatives to chemical herbicides for sustainable weed management. Previous study found that cultures of the pathogenic fungus *Colletotrichum gloeosporioides* BWH-1 showed phytotoxic activity. This study further isolated the major phytotoxin from cultures of the strain BWH-1 using bioactivity-guided isolation, by puncturing its host plant for an activity test and analyzing on the HPLC-DAD-3D mode for a purity check. Then, the active and pure phytotoxin was characterized as a dirhamnolipid (Rha-Rha-C10-C10) using the NMR, ESIMS, IR and UV methods. The herbicidal activity of dirhamnolipid was evaluated by the inhibition rate on the primary root length and the fresh plant weight of nine test plants, and the synergistic effect when combining with commercial herbicides. Dirhamnolipid exhibited broad herbicidal activity against eight weed species with IC_50_ values ranging from 28.91 to 217.71 mg L^−1^ and no toxicity on *Oryza sativa*, and the herbicidal activity could be synergistically improved combining dirhamnolipid with commercial herbicides. Thus, dirhamnolipid that originated from *C. gloeosporioides* BWH-1 displayed the potential to be used as a bioherbicide alone, or as an adjuvant added into commercial herbicides, leading to a decrease in herbicides concentration and increased control efficiency.

## 1. Introduction

Weeds compete with agricultural crops for water and nutrients, severely reducing crop yields, threatening the safety of world food production, and are one of the most serious agrarian pests that must be controlled. Strategies for weeds control can vary, including mechanical pulling, physical burial, cultural rotation, etc., but for many years the control of weeds has relied mainly on chemical herbicides [1,2]. However, the long-term and large-scale use of chemical herbicides has brought us many serious problems, especially the rapid evolution of resistant weed populations, and the substantial accumulation of herbicide residues in ecosystems that ultimately endanger human health [3]. Consequently, sustainable weed control strategies based on the use of natural products have become safer alternatives to chemical herbicides [1,4].

Bioherbicides are natural products originated from organisms or their metabolites with phytotoxic activity, which are more ecofriendly and more susceptible to degradation than chemical herbicides [5]. Only thirteen bioherbicides are currently available on the global market, while the global demands for bioherbicides continue rising due to the increase of public concern about the environmental safety of herbicides, the elimination of chemical herbicides using natural herbicides driven by legislation, and the need to improve weeds control efficiency [2]. 

Therefore, accelerating the discovering and development of bioherbicides has important practical value and far-reaching significance. Generally, the sources of bioherbicides are primarily microbial metabolites, especially phytotoxic metabolites produced by plant pathogenic fungi [1]. 

The plant pathogenic fungi belonging to the genus *Colletotrichum* usually cause serious anthracnose disease in a wide range of hosts worldwide, and the main symptoms of the affected parts are rapid necrosis, sunken and decay [6]. For this reason, species belonging to this genus have been utilized to identify and evaluate their phytotoxic metabolites for bioherbicide use. Recently, Masi et al. [7,8,9] carried out extensive studies on many species of the genus *Colletotrichum* for discovering potential active metabolites leading to the isolation of phytotoxins, among them colletopyrandione and colletochlorins. These results show that this genus *Colletotrichum* has great potential in discovering phytotoxins with potential herbicidal activity.

Previously, we isolated the pathogenic fungus *Colletotrichum gloeosporioides* from anthracnose disease-infected Bawanghua (*Hylocereus undatus*) in China [10], and named it as BWH-1. A previous study found that the cultures of this strain BWH-1 show phytotoxic activity when puncturing its host plant. Thus, we further investigated the phytotoxic metabolite produced by the strain BWH-1 for bioherbicide use in this study. Here, we isolated, purified and characterized the major phytotoxin from the cultures of *Colletotrichum gloeosporioides* BWH-1. Then, the herbicidal activity and synergistic effect that are combined with commercial herbicides of the target phytotoxin, were evaluated. Additionally, preliminary investigation on the mechanism of its herbicidal activity was also reported.

## 2. Results and Discussions

**Figure 1:**
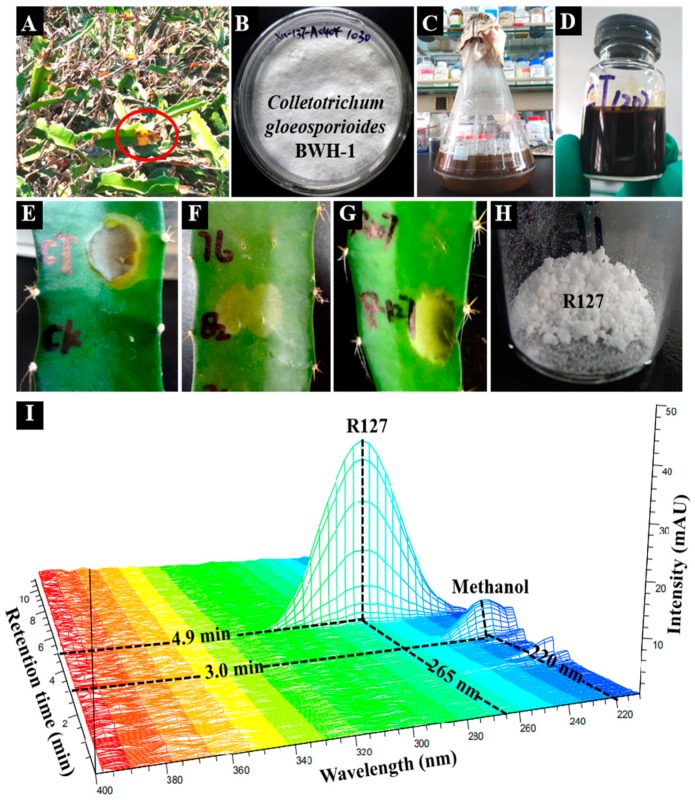
Bioactivity-guided isolation of the major phytotoxin produced by *Colletotrichum gloeosporioides* BWH-1. (**A**) Anthracnose disease of Bawanghua (*Hylocereus undatus*); (**B**) the pathogenic fungus *C. gloeosporioides* BWH-1; (**C**) liquid cultures of the strain BWH-1; (**D**) ethyl acetate (EtOAc) crude extracts of the liquid cultures; (**E**) puncture bioassay of EtOAc crude extracts; (F) puncture bioassay of fraction 82 isolated from EtOAc crude extracts; (**G**) puncture bioassay of fraction R127 isolated from fraction 82; (**H**) white crystals of R127 after purification; (**I**) purity check of R127 (265nm, 4.9min) by HPLC-DAD-3D chromatogram.

**Figure 2:**
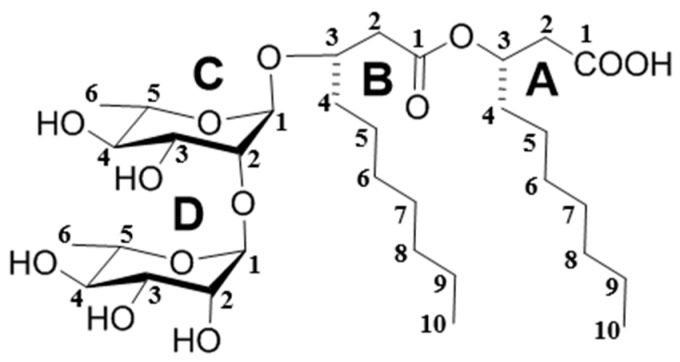
Structure of dirhamnolipid (Rha-Rha-C10-C10) identified from the pathogenic fungus *Colletotrichum gloeosporioides* BWH-1

As shown in Figure 1, bioactivity-guided isolation gave the target phytotoxin R127 (final yield 0.93 mg/L), which is described in details in the Materials and Methods section. The whole isolation process included ethyl acetate (EtOAc) crude extracts from liquid cultures of the strain BWH-1, following column chromatography fractions which were tested by puncture bioassay and a subsequent purity check by HPLC-DAD-3D chromatogram [11]. The structure of obtained major phytotoxin R127 was identified as a dirhamnolipid (Rha-Rha-C10-C10) (Figure 2) by comparison of NMR, ESI-MS, FT-IR and UV data, (this data is listed in the Materials and Methods section; spectra is placed in the Supplementary Materials, Figures S1–11), with those published by Sharma et al., which was previously found in the rizosphere of *Pseudomonas* [12]. Therefore, the major phytotoxin produced by the pathogenic fungus *C. gloeosporioides* BWH-1 is identified as a dirhamnolipid.

Interestingly, rhamnolipids are a group of biosurfactants including various monorhamnolipids and dirhamnolipids which are usually formed by one or two rhamnose molecules attached to one or two fatty acids of saturated or unsaturated alkyl chains between C_8_ and C_12_ [13,14]. They are generally produced by some bacteria species of *Pseudomonas aeruginosa* and *Burkholderia malleior* [15], and have become very promising bioactive molecules in recent years due to their novel structures, diverse bioactivities, high biodegradability, and that they can be produced from renewable resources [16]. To the best of our knowledge, this is the first report of the isolation of a dirhamnolipid from a fungus, and also the first report of a dirhamnolipid isolated from *C. gloeosporioides*. These results suggest that rhamnolipids are not only present in the metabolites of certain strains of bacteria, but *C. gloeosporioides* BWH-1 can also be used as a new strain for production of the promising bioactive dirhamnolipid. 

Moreover, dirhamnolipid was previously reported to possess strong insecticidal activities against green peach aphid [17] and mosquito pupae [18]. However, to date, there are few reports on its herbicidal activity. Thus, we conducted research on the herbicidal activity of dirhamnolipid in this study.

The herbicidal activity of dirhamnolipid was evaluated by inhibiting the primary root length and fresh plant weight of nine test plants (eight weed species and *Oryza sativa*). Dirhamnolipid exhibited broad herbicidal activity against the eight weed species with half of the maximal inhibitory concentration (IC_50_) values ranging from 28.91 to 145.23 mg L^−1^ on the primary root length and 50.07 to 217.71 mg L^−1^ on the fresh plant weight, while no toxicity was found against *O. sativa* (Table 1). Compared to the herbicidal activity of berberine [4], the dirhamnolipid showed relatively weak herbicidal activity, which may help to ensure the safety of dirhamnolipid application. 

Thus, the dirhamnolipid that was originated from *C. gloeosporioides* BWH-1 displayed the potential to be used as a bioherbicide.

**Figure 3:**
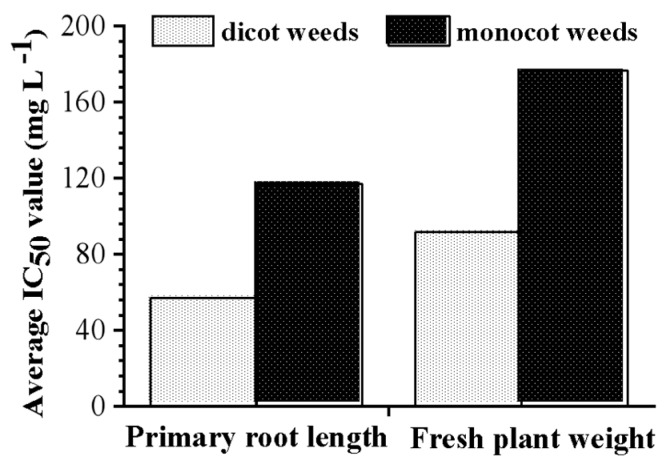
Average IC_50_ values of this dirhamnolipid on dicot weeds and monocot weeds

In addition, the average IC_50_ value of dirhamnolipid on the primary root length of tested dicot weeds (56.93 mg L^−1^) was two times lower than that of monocot weeds (117 mg L^−1^) (Figure 3). Meanwhile, the average IC_50_ value of dirhamnolipid on the fresh plant weight of dicot weeds (91.61 mg L^−1^) was 1.93 times lower than that of monocot weeds (176.87 mg L^−1^) (Figure 3). These results indicate that the dirhamnolipid showed better herbicidal activity against dicot weeds than monocot weeds.

According to Yan et al. [14], rhamnolipids combined with laurel oil have a synergistic effect against *Alternaria alternata*, leading to the decrease of fungicides concentration and increase of control efficiency. So, the herbicidal synergy of dirhamnolipid (DiRL) was evaluated when it combined with commercial herbicides, cyhalofop-butyl (CB) or penoxsulam (PEN), against four dicot weeds.

**Figure 4:**
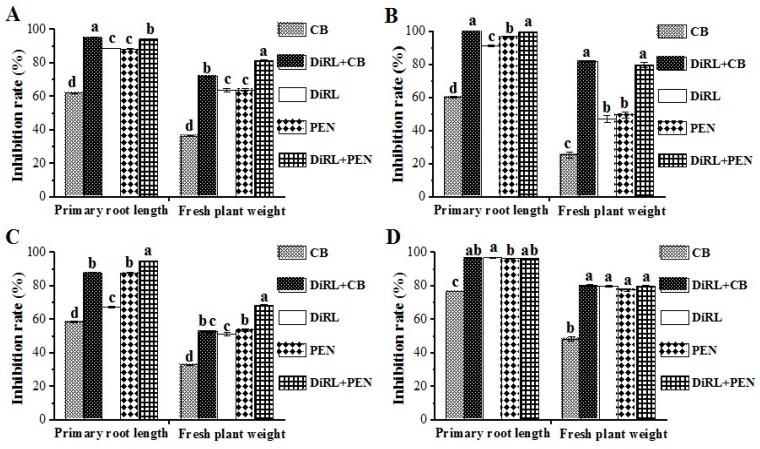
Inhibition rates of dirhamnolipid combined with cyhalofop-butyl or penoxsulam on the primary root length and fresh plant weight of four test dicot weeds at the concentration of 100 mg L^−1^ (7d). (**A**) *B. pilosa*; (**B**) *M. micrantha*; (**C**) *A. retroflexus*; (**D**) *A. Conyzoides*. CB: Cyhalofop-butyl. DiRL + CB: Dirhamnolipid combined with cyhalofop-butyl. DiRL: Dirhamnolipid. PEN: Penoxsulam. DiRL + PEN: Dirhamnolipid combined with penoxsulam. Same letter represent values that are not significantly different at the 0.05 probability level according to Duncan’s multiple range test.

As shown in Figure 4A, the treatment DiRL + CB reduces the primary root length and fresh plant weight of *B. pilosa* by 94.79% and 71.74% respectively, which were significantly higher than that caused by either DiRL (88.50% and 63.58%., respectively) or CB (61.82% and 36.13%, respectively) alone. Meanwhile, using DiRL + PEN reduces the primary root length and fresh plant weight of *B. pilosa* by 94.02% and 81.42%, respectively, which were also significantly higher than that caused by either DiRL (88.50% and 63.58%, respectively) or PEN (88.22% and 63.81%, respectively) alone. In parallel, similar reductions were observed inhibiting *M. micrantha* (Figure 4B) and *A. retroflexus* (Figure 4C). These results suggest that both combinations exhibit significant synergistic effects against the three dicot weeds. Additionally, the use of DiRL+CB or DiRL+PEN shows additive effects against *A. Conyzoides* (Figure 4D) because the inhibition rates of combinations were not significantly different to being used alone. These results indicate that the herbicidal activity of dirhamnolipid could be synergistically improved when combining with commercial herbicides leading to decreased herbicides concentration and increased control efficiency.

Importantly, DiRL + CB shows no antagonism and significantly improves the herbicidal activity against the four tested dicot weeds compared to using CB alone (Figure 4). In contrast, Ottis et al. [19] reports that CB is antagonized by some rice herbicides, while Kim et al. [20] report that CB is an effective herbicide for monocot weeds control, but inefficient for dicot weeds control. Our results suggest that the combination of dirhamnolipid with cyhalofop-butyl could be used to improve the control efficiency of dicot weeds.

Mechanisms involved in the synergistic effect of rhamnolipids combined with laurel oil was supported by Yan et al. [14], that rhamnolipids interact and dehydrate the plasma membrane of *Alternaria alternata*, and then allow essential oils to penetrate into cytoplasm more easily, so that the combination can exert a greater inhibitory effect than if it was used alone. So, Scanning Electron Microscope (SEM) observation on the root tip mature area of *B. pilosa* treated with different concentrations of dirhamnolipid was employed to investigate preliminarily whether the herbicidal activity of dirhamnolipid had a similar mechanism of action.

**Figure 5:**
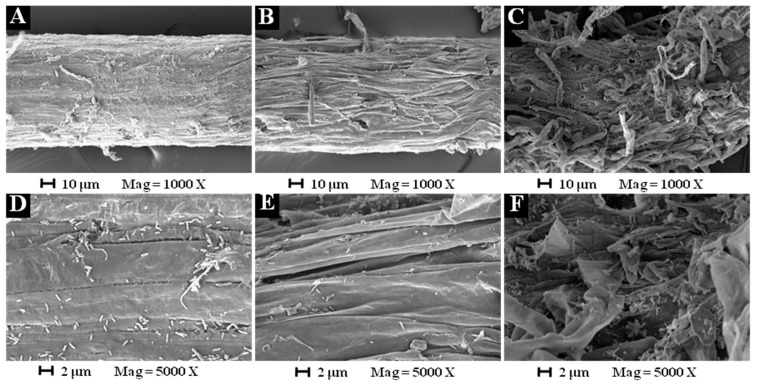
Scanning Electron Microscope (SEM) observation on the root tip mature area of *Bidens pilosa*. (**A**,**D**) Untreated control (magnification, ×1000 and ×5000); (**B**,**E**) treated with 500 mg L^−1^ dirhamnolipid (magnification, ×1000 and ×5000); (**C**,**F**) treated with 1000 mg L^−1^ dirhamnolipid (magnification, ×1000 and ×5000).

Via SEM observations, the regular morphology with full shape was obtained on the root tip mature area of *B. pilosa* with no treatment of the dirhamnolipid, whilst irregular morphology with severe dehydration was observed with the treatment of 500 mg L^−1^ or 1000 mg L^−1^ dirhamnolipid (Figure 5). These morphology changes preliminarily suggest that the herbicidal activity of dirhamnolipid could possess the similar mechanism that interacted with and dehydrated the target part of weeds, and thus caused its death. However, further investigations on the specific mechanism of the action of any dirhamnolipid should be carried out.

## 3. Materials and Methods 

### 3.1. General Experimental Procedures

^1^H and ^13^C NMR spectra were recorded at 600 MHz and 150 MHz in MeOD on a Bruker Avance 600 spectrometer (Bruker, Karlsruhe, Germany). The same solvent was used as an internal standard. ESI-MS analysis was conducted using a Waters LC-MS system (Waters, MA, USA) with an ACQUITY UPLC system coupled to the Waters Q-Tof Premier high-resolution mass spectrometer. FT-IR spectrum was obtained on a Nicolet 6700 FT-IR System (Thermo Scientific, MA, USA). Column chromatography was performed using silica gel (200–300 mesh, Qingdao Marine Chemical Factory, Qingdao, China) and ODS (SP-120-40/60-ODS-RPS, Daiso Co., Ltd., Osaka, Japan), respectively. Analytical thin layer chromatography (TLC) was performed on silica gel (GF254, Qingdao Marine Chemical Factory, Qingdao, China) plates. An HPLC-DAD-3D chromatogram was made on an Agilent 1290 Infinity II HPLC system (Agilent Technologies, Inc., CA, USA) equipped with a DAD detector.

### 3.2. Strain and Liquid Culture

The strain *Colletotrichum gloeosporioides* BWH-1 used in this study was isolated from anthracnose disease-infected Bawanghua (*Hylocereus undatus*) in China, as reported in our previous paper [10]. It was maintained on PDA plates at 28 °C for seven days and then twenty fresh mycelium pieces (0.5 cm × 0.5 cm) were inoculated into 500 mL Czapek-dox seed medium at 28 °C, 160 rpm for seven days. The seed culture (100 mL) was then transferred into 2500 mL fresh Czapek-dox medium for further culture at 28 °C, 160 rpm for 14 days.

### 3.3. Bioactivity-guided Isolation and Purification

The liquid cultures (10 L) of the strain BWH-1 was centrifuged, and then the obtained supernatant was extracted with ethyl acetate (EtOAc) to obtain dark brown crude extracts (10.1 g) after evaporation of the solvent. The EtOAc crude extracts were separated by column chroma-tography over silica gel (200–300 mesh, Qingdao Marine Chemical Factory, Qingdao, China) with gradient elution of CHCl_2_/MeOH and 105 fractions were obtained. Monitored by TLC (GF254, Qingdao Marine Chemical Factory, Qingdao, China), these 105 fractions were divided into eight homogenous groups (fraction 12, 20, 31, 43, 61, 76, 82 and 105). The fraction 82 (2.5 g) showed phytotoxic activity, and was further separated by column chromatography over ODS (SP-120-40/60-ODS-RPS, Daiso Co., Ltd., Osaka, Japan) with gradient elution of H_2_O/MeOH, and 130 fractions were obtained. Monitored by TLC, 130 fractions were divided into 10 homogenous groups (fraction R8, R25, R37, R65, R69, R84, R100, R102, R107 and R127). The fraction R127 (1.2 g) showed phytotoxic activity, and was further purified by recrystallization (final yield 0.93 mg/L). The purity of bioactive fraction R127 was checked by the HPLC-DAD-3D chromatogram (Agilent 1290 Infinity II, Agilent, CA, United States) [11]. The phytotoxic activity of crude extracts and all fraction groups were tested by puncture bioassay on the host plant [9]. See Figure 1 for the entire isolation and purification procedures.

### 3.4. Structure Characterization

Dirhamnolipid (Figure 2), Rha-Rha-C10-C10. White crystals; UV λ_max_ nm (log ε) 265 nm; IR ν_max_ 3336, 2924, 2856, 1734, 1648, 1459, 1118 and 1052 cm^−1^; ^1^H NMR (600 MHz, MeOD) *δ*: 5.26 [1H, pentet, *J* = 6.0, (A)H-3], 4.94 [1H, d, *J* =1.2, (C)H-1], 4.90 [1H, d, *J* =1.8, (D)H-1], 4.06 [1H, pentet, *J* = 6.0, (B)H-3], 3.98 [1H, dd, *J* = 3.0, 1.5, (D)H-2], 3.67-3.76 [5H, m, (C)H-2,3,5, (D)H-3,5], 3.40 [1H, t, *J* = 9.6, (C)H-4], 3.33 [1H, t, *J* = 9.6, (D)H-4], 2.59 [3H, m, (A)H-2, (B)H-2a], 2.50 [1H, dd, *J* = 15.0, 6.0, (B)H-2b], 1.64 [2H, d, *J* = 6.6, (A)H-4], 1.57 [2H, d, *J* = 7.2, (B)H-4], 1.33 [20H, m, (A)H-5,6,7,8,9, (B)H-5,6,7,8,9], 1.27 [3H, d, *J* = 6, (C)H-6], 1.25 [3H, d, *J* = 6, (D)H-6], 0.92 [6H, t, *J* = 7.2, (A)H-10, (B)H-10]; ^13^C NMR (150 MHz, MeOD) δ: 174.50 [(A)C-1], 172.63[(B)C-1], 104.32 [(D)C-1], 99.34 [(C)C-1], 80.55 [(C)C-2], 75.55 [(B)C-3], 74.38 [(C)C-4], 73.96 [(D)C-4], 72.50 [(A)C-3], 72.37 [(D)C-3], 72.02 [(C)C-3, (D)C-2], 70.38 [(D)C-5], 70.26 [(C)C-5], 41.39 [(B)C-2], 40.04 [(A)C-2], 35.14 [(A)C-4], 34.39 [(B)C-4], 33.05, 32.98, 30.81, 30.46, 30.40, 30.35, 26.28, 25.93, 23.76, 23.76, 18.16 [(C)C-6], 18.11 [(D)C-6], 14.52 [(A)C-10, (B)C-10]; ESIMS (–) *m*/*z*: 649.3323 [M–H]^−^ (calculated for C_32_H_58_O_13_, 650.3877). These data are consistent with the published data [12].

### 3.5. Evaluation of Herbicidal Activity

The herbicidal activity of dirhamnolipid was evaluated according to the described method [4] by inhibiting the primary root length and fresh plant weight of nine test plants, including *Oryza sativa* and eight test weeds. The seeds of dicot weeds (*Ageratum conyzoides*, *Celosia argentea*, *Bidens pilosa*, *Mikania micrantha*, *Capsella bursa-pastoris* and *Amaranthus retroflexus*) were provided by the research group of Prof. Lijuan Zhou, South China Agricultural University. The seeds of monocot weeds (*Alopecurus aequalis* and *Echinochloa crusgalli*) were provided by Dr. Lanlan Sun, Henan Academy of Agricultural Sciences. All of the tested seeds used in this research were first immersed in 1.0% (*v*/*v*) sodium hypochlorite solutions for 10 min, killing fungi and bacteria. 

Then the sterilized seeds were washed three times by sterilized water and immersed in water for 1–3 days at room temperature until germinated. The germinated seeds were immediately placed in a refrigerator at 4 °C, waiting for use. Dirhamnolipid was first dissolved in acetone and then diluted with sterilized water to get a series of concentrations from 50 to 1000 mg L^−1^. Different concentrations of dirhamnolipid solutions were introduced into six-well cell culture plates (5 mL/well) which filled with 2 mm diameter glass beads that mechanically supported seedlings growth. Sterilized water containing the same amount of acetone was used as a negative control. Berberine [4] was used as the positive control. Ten germinated seeds were placed in each well of the culture plates, and then each plate was covered with polyethylene wrapping film which was perforated with several little holes for ventilation. Then the ready culture plates were placed in a growth chamber calibrated to provide 12 h light/12 h darkness at 25 ± 2 °C. A completely randomized design was selected, and all treatments were replicated at least three times. After seven days of treatment, the fresh plant weight of each seedling was weighed, and the primary root length of each photographed seedling was measured on the software Image Pro Plus 6.0 (Media Cybernetics, Inc., Rockville, MD, USA). The inhibition rate on the primary root length and fresh plant weight of all tested plants was calculated by the following formula:

(1) $$\mathrm{Inhibition}\;\mathrm{rate}\;\left(\%\right)=\frac{\mathrm{negative}\;\mathrm{control}\;–\;\mathrm{treatment}}{\mathrm{negative}\;\mathrm{control}}\times 100$$

### 3.6. Evaluation of Synergistic Effect

The synergistic effect was evaluated using dirhamnolipid (DiRL) combined with commercial herbicides, cyhalofop-butyl (CB) (Sigma, CAS: 122008-85-9) or penoxsulam (PEN) (Sigma, CAS: 219714-96-2), against four dicot weeds (*B. pilosa*, *M. micrantha*, *A. retroflexus* and *A. conyzoides*) [14,21]. Seeds of the four tested weeds were pretreated as described above. The concentrations of test solutions were treated as follows: (1) Sterilized water used as negative control; (2) DiRL at 100 mg L^−1^; (3) CB at 100 mg L^−1^; (4) PEN at 100 mg L^−1^; (5) DiRL + CB at 100 mg L^−1^; (6) DiRL + PEN at 100 mg L^−1^. A similar set of experiments were conducted as described above. All treatments were repeated at least three times. The inhibition rate on the primary root length and fresh plant weight of four tested dicot weeds was investigated at seven days after treatment.

### 3.7. Microscopy Observation

The mechanism of the herbicidal activity of dirhamnolipid was preliminarily investigated on the root tip mature area of *B. pilosa* by scanning electron microscopy (SEM) observations [4]. The roots of seven days old *B. pilosa* seedlings were immersed in the 500 mg L^−1^ dirhamnolipid and 1000 mg L^−1^ dirhamnolipid solutions respectively for seven days. Seedlings were immersed in water as untreated control. The SEM samples were processed using common methods [22]. The processed samples were observed by XL-30-ESEM (FEI, Eindhoven, Netherlands).

### 3.8. Statistical Analysis

The inhibition rates of the primary root length and fresh plant weight were analyzed using IBM SPSS 20.0 software. All data were presented as the means ± SE of at least three independent experiments. One-way Analysis of Variance (ANOVA) analyses and Duncan’s multiple range test were used to detect statistical significance, and differences were considered significant when *p* < 0.05.

## 4. Conclusions

In summary, dirhamnolipid was identified as the major phytotoxin produced by the pathogenic fungus *C. gloeosporioides* BWH-1 for the first time. Moreover, dirhamnolipid exhibited broad herbicidal activity which could be synergistically improved when combining with commercial herbicides. These findings indicate that dirhamnolipid has the potential to be used as a bioherbicide alone, or as an adjuvant added into commercial herbicides, leading to decrease the herbicides concentration and to increase control efficiency.

## Figures and Tables

**Table 1:** Inhibition rate (%) and IC_50_ values of dirhamnolipid on the primary root length and fresh plant weight of the nine test plants (7d) ^a^.

Test Plants		Inhibition Rate (%) ^b^					IC_50_ (mg L^−1^) ^c^
		50 mg L^−1^	100 mg L^−1^	250 mg L^−1^	500 mg L^−1^	1000 mg L^−1^	
*Ageratum Conyzoides*	L	70.11 ± 0.02d	85.78 ± 0.08c	92.64 ± 0.14b	100 ± 0.00a	100 ± 0.00a	28.91
	W	48.99 ± 0.49c	71.18 ± 0.85b	73.34 ± 2.10b	100 ± 0.00a	100 ± 0.00a	55.95
*Celosia argentea*	L	52.17 ± 0.08c	88.00 ± 0.02b	100 ± 0.00a	100 ± 0.00a	100 ± 0.00a	47.24
	W	57.10 ± 0.10c	69.11 ± 0.10b	100 ± 0.00a	100 ± 0.00a	100 ± 0.00a	50.07
*Bidens pilosa*	L	51.94 ± 0.02d	82.98 ± 0.02c	92.67 ± 0.06b	100 ± 0.00a	100 ± 0.00a	47.34
	W	38.09 ± 0.06d	55.68 ± 0.14c	76.51 ± 0.18b	100 ± 0.00a	100 ± 0.00a	79.56
*Mikania micrantha*	L	40.59 ± 1.62d	82.37 ± 0.71c	94.27 ± 0.18b	100 ± 0.00a	100 ± 0.00a	57.13
	W	8.54 ± 2.86d	63.11 ± 1.10c	64.18 ± 0.81c	87.04 ± 0.40b	100 ± 0.00a	123.72
*Capsella bursa-pastoris*	L	51.48 ± 0.18d	51.28 ± 0.06d	70.87 ± 0.03c	75.51 ± 0.10b	92.85 ± 0.06a	65.68
	W	38.34 ± 1.14c	41.64 ± 1.86c	77.05 ± 0.57b	78.25 ± 0.29b	89.51 ± 0.19a	98.25
*Echinochloa crusgalli*	L	31.51 ± 0.11e	58.21 ± 0.09d	74.50 ± 0.04c	86.16 ± 0.06b	90.91 ± 0.03a	88.77
	W	27.08 ± 0.07d	50.97 ± 0.06c	68.16 ± 0.41b	68.86 ± 0.26ab	69.46 ± 0.47a	136.03
*Amaranthus retroflexus*	L	31.82 ± 0.10e	52.53 ± 0.04d	68.66 ± 0.03c	96.26 ± 0.04b	100 ± 0.00a	95.29
	W	21.21 ± 0.26e	42.17 ± 0.10d	64.96 ± 0.10c	74.74 ± 0.10b	100 ± 0.00a	142.10
*Alopecurus aequalis*	L	20.04 ± 0.34e	45.74 ± 0.25d	60.79 ± 0.11c	76.62 ± 0.12b	95.77 ± 0.01a	145.23
	W	24.09 ± 1.02d	25.97 ± 0.63d	56.90 ± 0.69c	71.52 ± 0.18b	76.46 ± 0.22a	217.71
*Oryza sativa*	L	13.20 ± 0.12d	21.34 ± 0.04c	38.58 ± 0.10b	38.67 ± 0.12b	54.13 ± 0.04a	779.44
	W	0.20 ± 0.04b	0.18 ± 0.03b	1.27 ± 0.90b	4.70 ± 0.06a	4.58 ± 0.33a	>1000

^a^ All treatments were replicated at least three times. ^b^ All data represent means ± SE. Values followed by same letter in the same line are not significantly different at the 0.05 probability level, according to Duncan’s multiple range test. ^c^ IC_50_ represents half of the maximal inhibitory concentration. L: Primary root length. W: Fresh plant weight.

## Supplementary Materials

The following supplementary materials are available online. Figure S1–S3. ^1^H NMR of the isolated major phytotoxin R127 in MeOD. Figure S4–S6. ^13^C NMR of the isolated major phytotoxin R127 in MeOD. Figure S7–S9. HSQC of the isolated major phytotoxin R127 in MeOD. Figure S10. ESI-MS of the isolated major phytotoxin R127. Figure S11. FT-IR of the isolated major phytotoxin R127. Figure S12. Inhibitory effect of dirhamnolipid against the 8 tested weeds (7d). Figure S13. Synergistic effects of dirhamnolipid combined with cyhalofop-butyl or penoxsulam on the 4 test dicot weeds at the concentration of 100 mg L^−1^ (7d). (**A**) *A. Conyzoides*; (**B**) *B. pilosa*; (**C**) *M. micrantha*; (**D**) *A. retroflexus*. CK: negative control. CB: cyhalofop-butyl. PEN: penoxsulam. DiRL: dirhamnolipid. DiRL + CB: dirhamnolipid combined with cyhalofop-butyl. DiRL + PEN: dirhamnolipid combined with penoxsulam.
