# Supplementary material for: Dirhamnolipid Produced by the Pathogenic Fungus Colletotrichum gloeosporioides BWH-1 and Its Herbicidal Activity
Source: Molecules. 2019 Aug 16;24(16):2969. doi: 10.3390/molecules24162969 (PMC6720573; doi:10.3390/molecules24162969)
Supplement: Supplementary file 1 [file molecules-24-02969-s001.pdf]

Supplementary Material

Dirhamnolipid Produced by the Pathogenic Fungus *Colletotrichum gloeosporioides* BWH-1 and Its Herbicidal Activity

Zhaolin Xu, Mengying Shi, Yongqing Tian, Pengfei Zhao, Yifang Niu, Meide Liao *

Key Lab of Natural Pesticides & Chemical Biology, Ministry of Education, Department of Pesticide Science, South China Agricultural University, Guangzhou, Guangdong, 510642, China; zhaolinxu2019@126.com (Z.X.); mengyingshi99@163.com (M.S.); tyq2006@scau.edu.cn (Y.T.); pengfeizhaostu@gmail.com (P.Z.); n826641070@163.com (Y.N.)

* Correspondence: liaomeide@scau.edu.cn; Tel.: +86 13610008987

Supplementary Information:

Characterization of the isolated major phytotoxin R127 (Figure S1-S11):

The structure of isolated major phytotoxin R127 was identified as dirhamnolipid (Rha-Rha-C10-C10) by comparison of the obtained data below with those published by Sharma et al., which was previously found in the rizosphere of *Pseudomonas* (Sharma et al., J. Nat. Prod., 2007).

Figure S1. ^{1}H NMR (full spectrum) of the isolated major phytotoxin R127 in MeOD.

Figure S2. ^{1}H NMR (expanded spectrum 1) of the isolated major phytotoxin R127 in MeOD.

Figure S3. ^{1}H NMR (expanded spectrum 2) of the isolated major phytotoxin R127 in MeOD.

Figure S4. ^{13}C NMR (full spectrum) of the isolated major phytotoxin R127 in MeOD.

Figure S5. ^{13}C NMR (expanded spectrum 1) of the isolated major phytotoxin R127 in MeOD.

Figure S6. ^{13}C NMR (expanded spectrum 2) of the isolated major phytotoxin R127 in MeOD.

Figure S7. HSQC (full spectrum) of the isolated major phytotoxin R127 in MeOD.

Figure S8. HSQC (expanded spectrum 1) of the isolated major phytotoxin R127 in MeOD.

Figure S9. HSQC (expanded spectrum 2) of the isolated major phytotoxin R127 in MeOD.

Figure S10. ESI-MS of the isolated major phytotoxin R127.

Figure S11. FT-IR of the isolated major phytotoxin R127.

Herbicidal activity of dirhamnolipid:

Figure S12. Inhibitory effect of dirhamnolipid against the 8 tested weeds (7d).

Figure S13. Synergistic effects of dirhamnolipid combined with cyhalofop-butyl or penoxsulam on the 4 test dicot weeds at the concentration of 100 mg L^{-1} (7d). (A) *A. Conyzoides*; (B) *B. pilosa*; (C) *M. micrantha*; (D) *A. retroflexus*. CK: negative control. CB: cyhalofop-butyl. PEN: penoxsulam. DiRL: dirhamnolipid. DiRL+CB: dirhamnolipid combined with cyhalofop-butyl. DiRL+PEN: dirhamnolipid combined with penoxsulam.

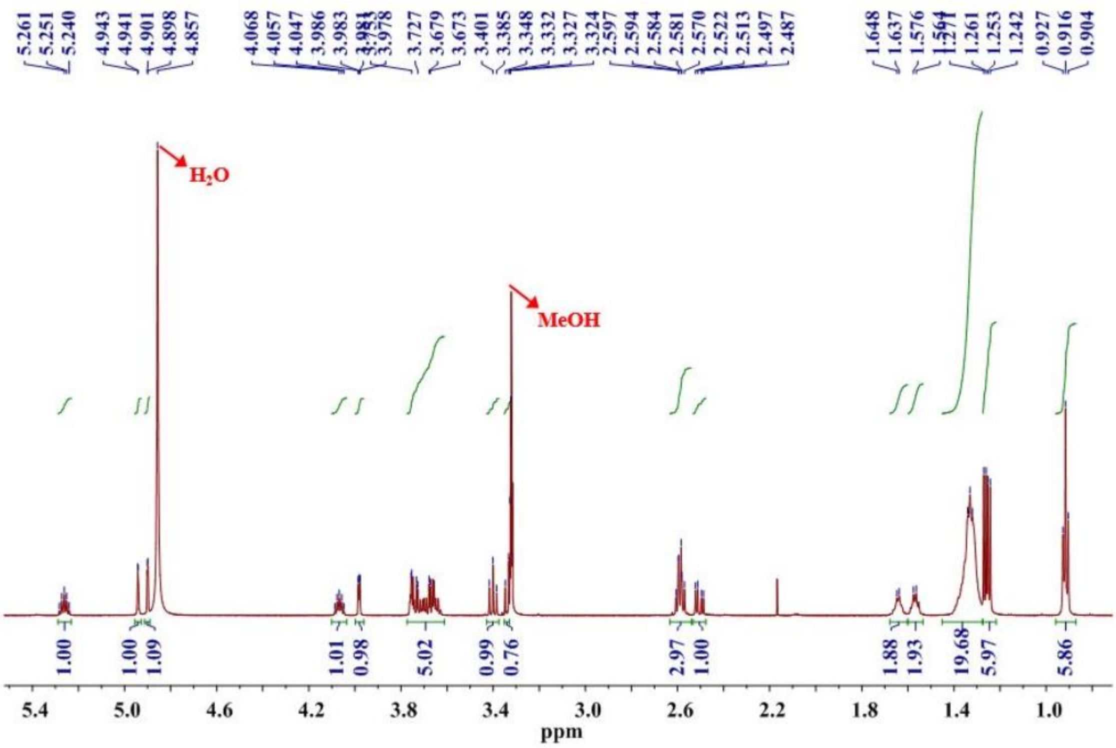

Figure S1. ^{1}H NMR (full spectrum) of the isolated major phytotoxin R127 in MeOD.

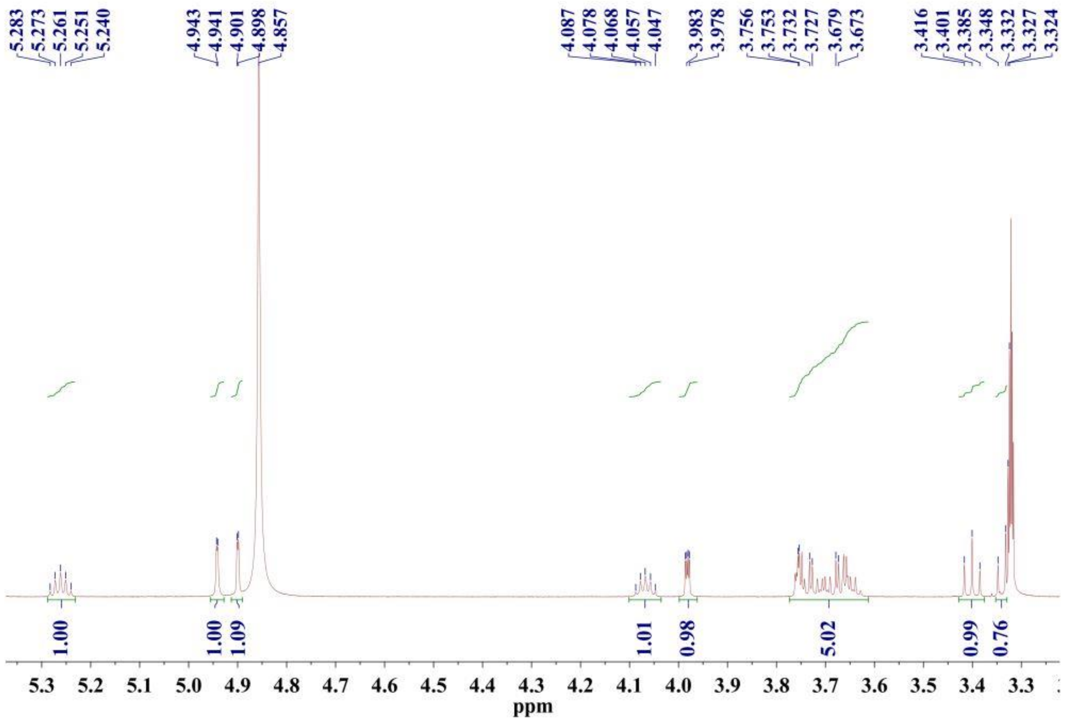

Figure S2. ^{1}H NMR (expanded spectrum 1) of the isolated major phytotoxin R127 in MeOD.

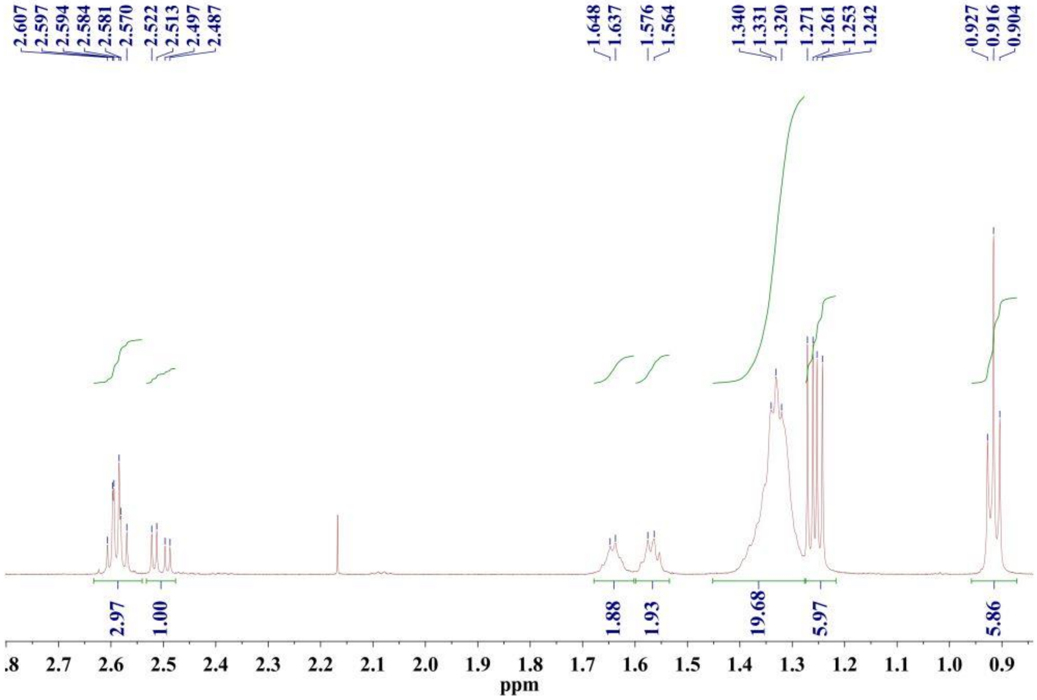

Figure S3. ^{1}H NMR (expanded spectrum 2) of the isolated major phytotoxin R127 in MeOD.

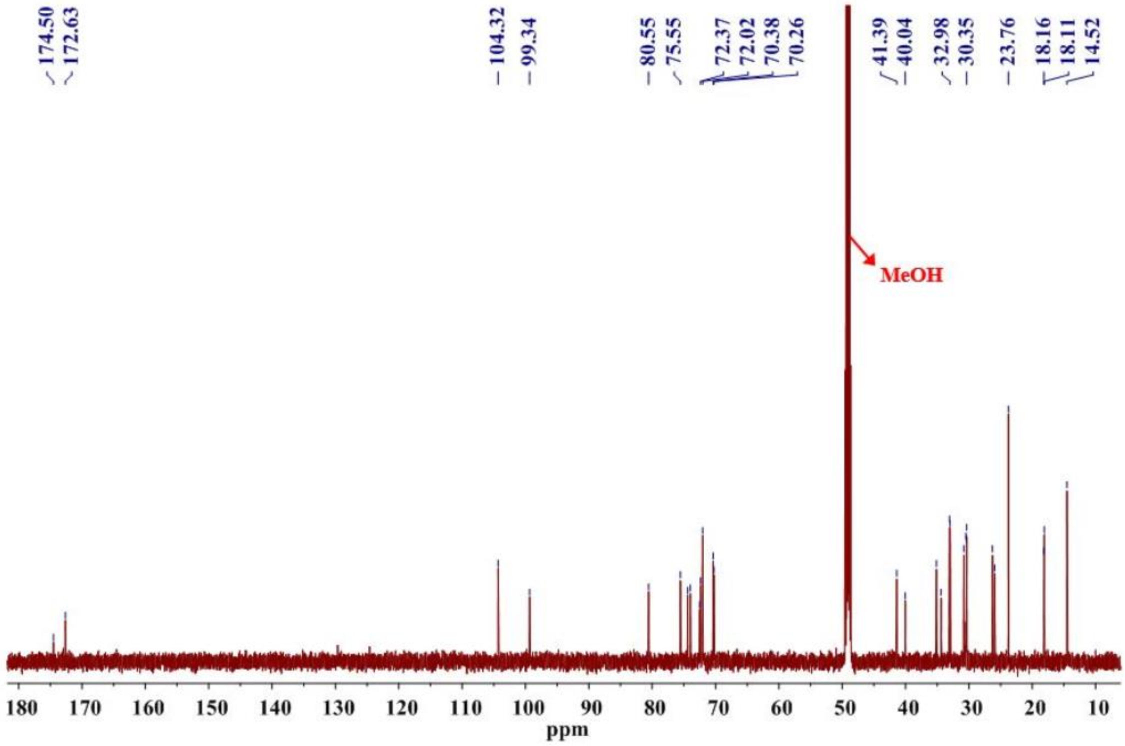

Figure S4. ^{13}C NMR (full spectrum) of the isolated major phytotoxin R127 in MeOD.

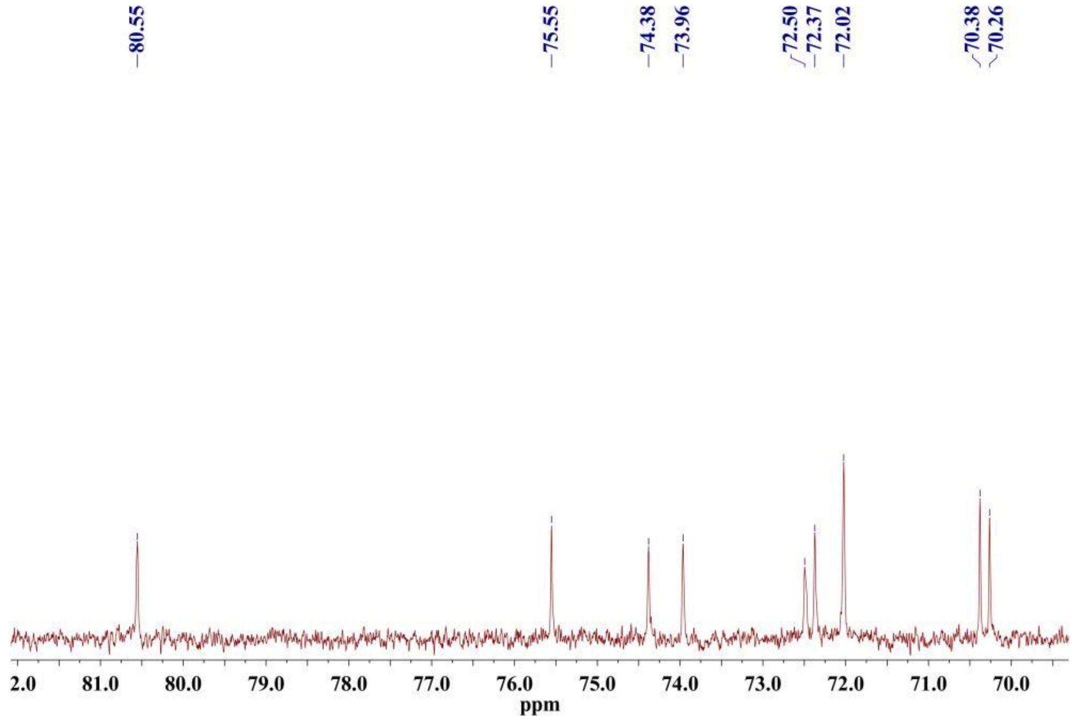

Figure S5. ^{13}C NMR (expanded spectrum 1) of the isolated major phytotoxin R127 in MeOD.

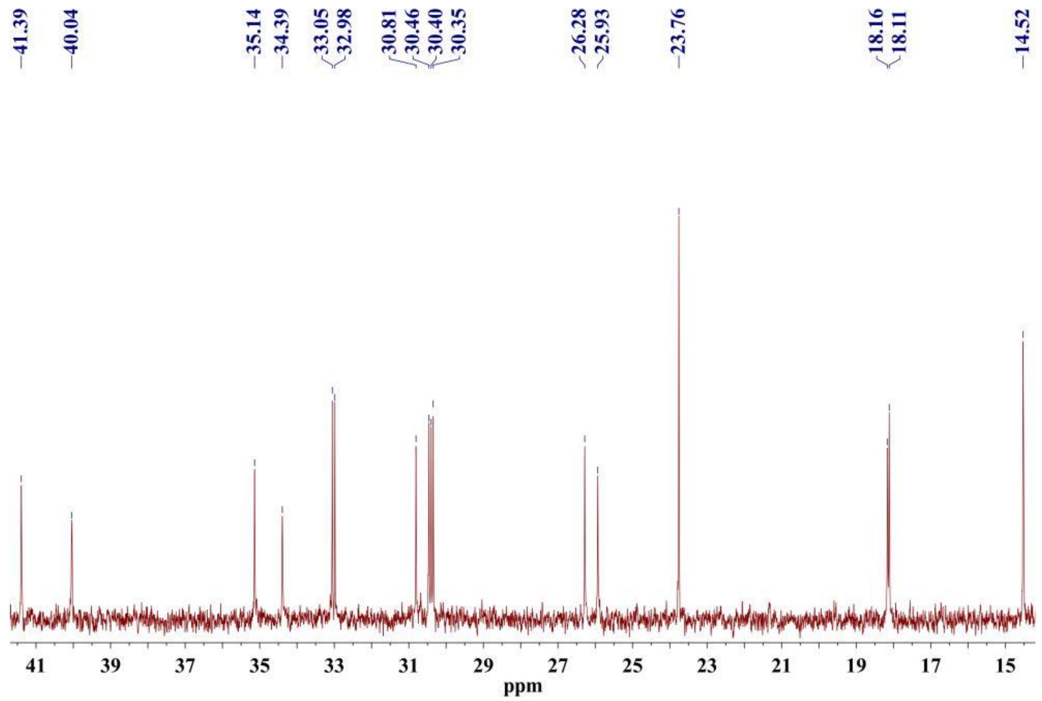

Figure S6. ^{13}C NMR (expanded spectrum 2) of the isolated major phytotoxin R127 in MeOD.

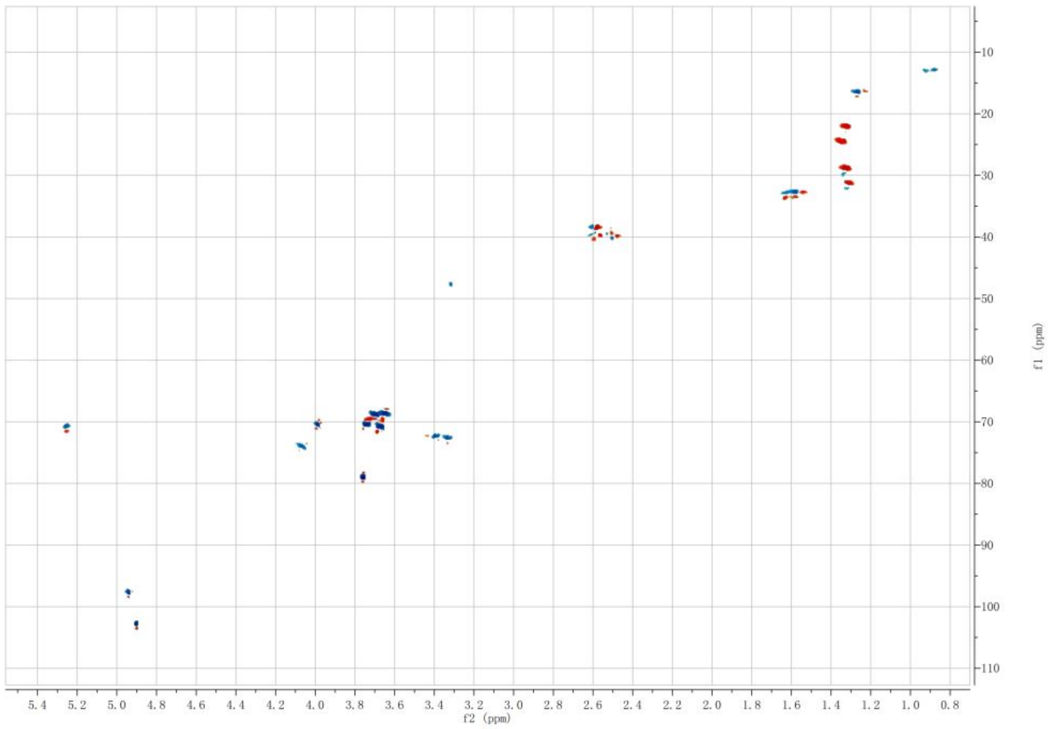

Figure S7. HSQC (full spectrum) of the isolated major phytotoxin R127 in MeOD.

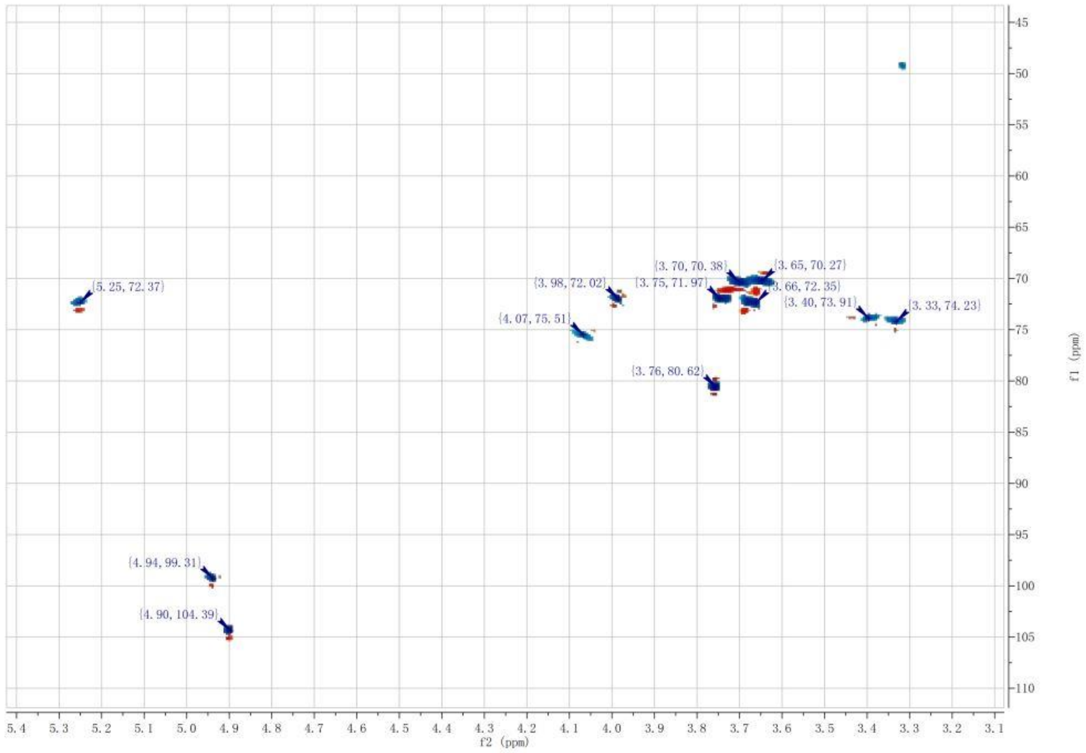

Figure S8. HSQC (expanded spectrum 1) of the isolated major phytotoxin R127 in MeOD.

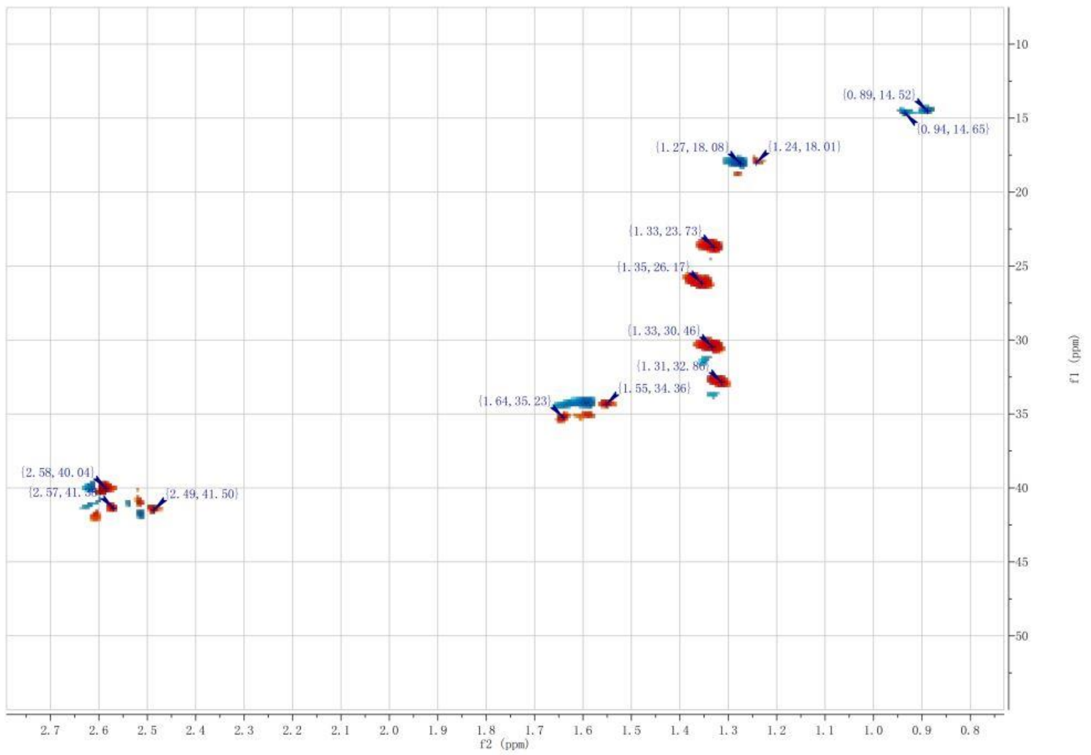

Figure S9. HSQC (expanded spectrum 2) of the isolated major phytotoxin R127 in MeOD.

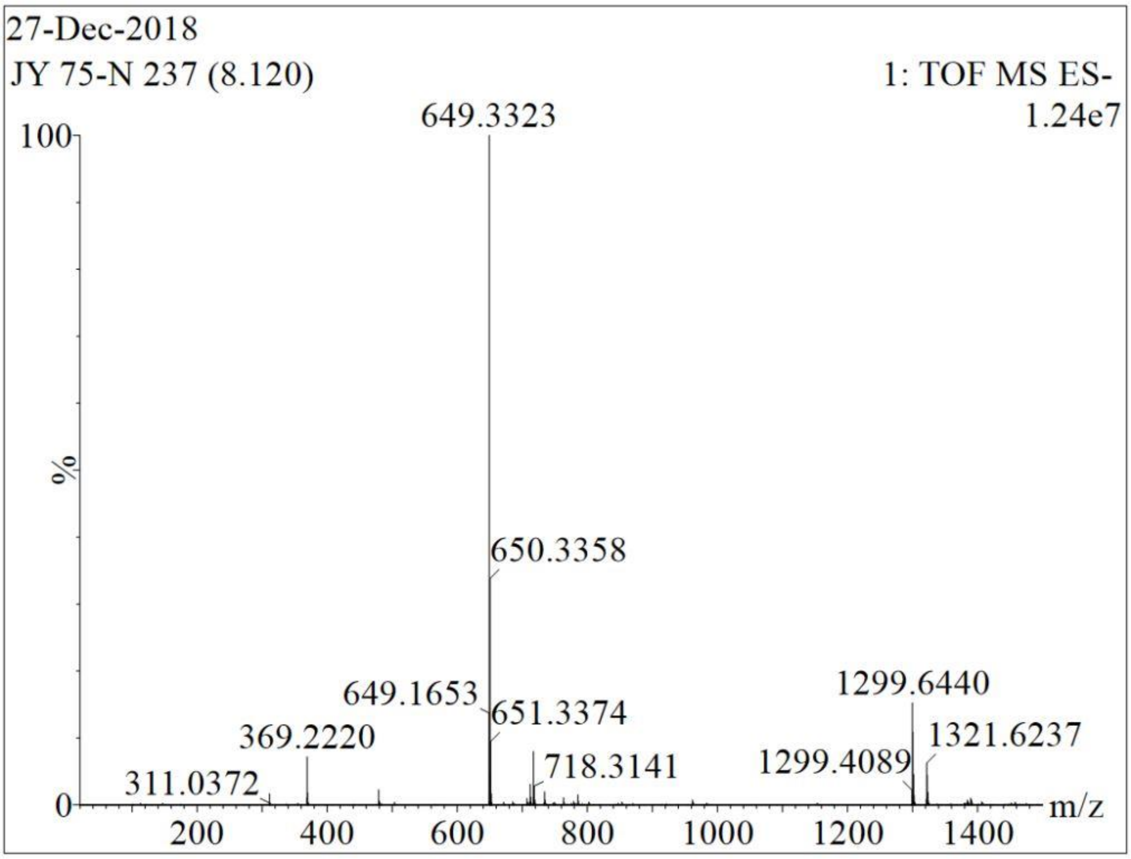

Figure S10. ESI-MS of the isolated major phytotoxin R127.

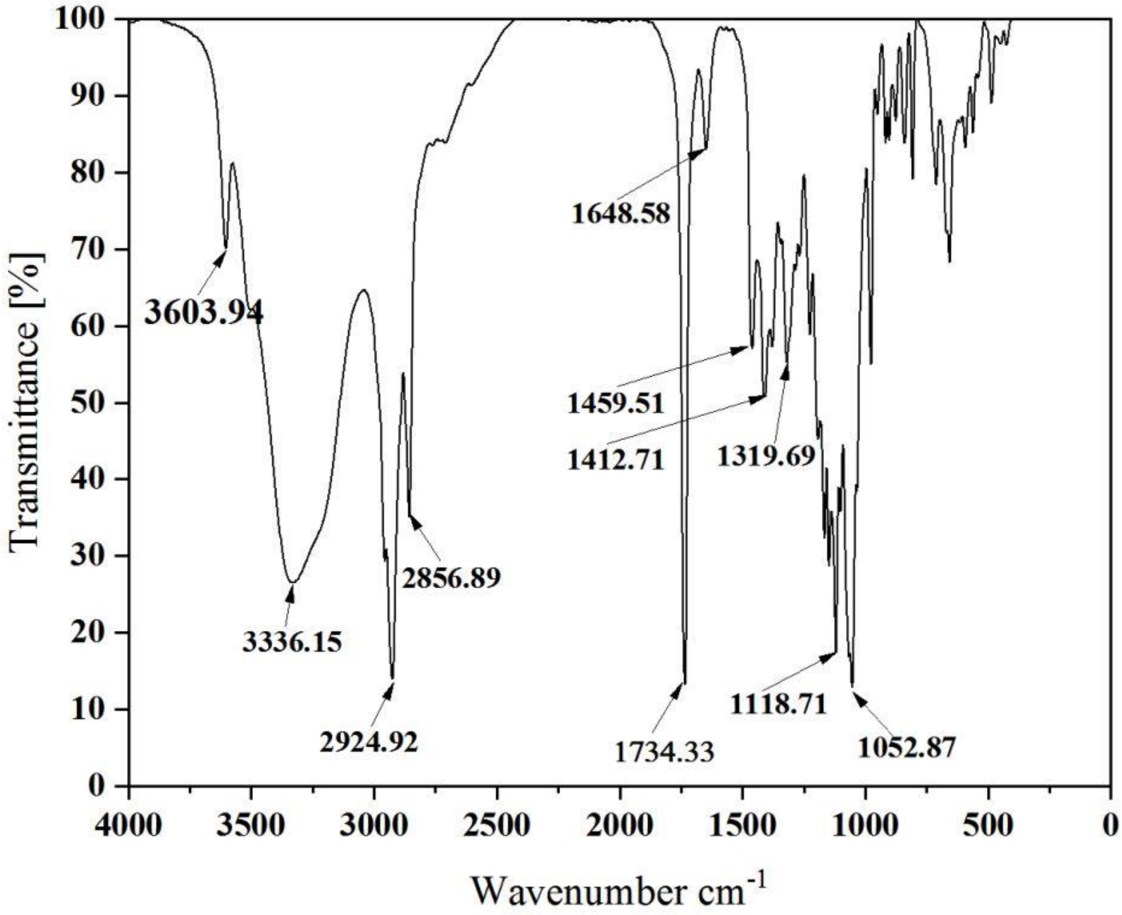

Figure S11. FT-IR of the isolated major phytotoxin R127.

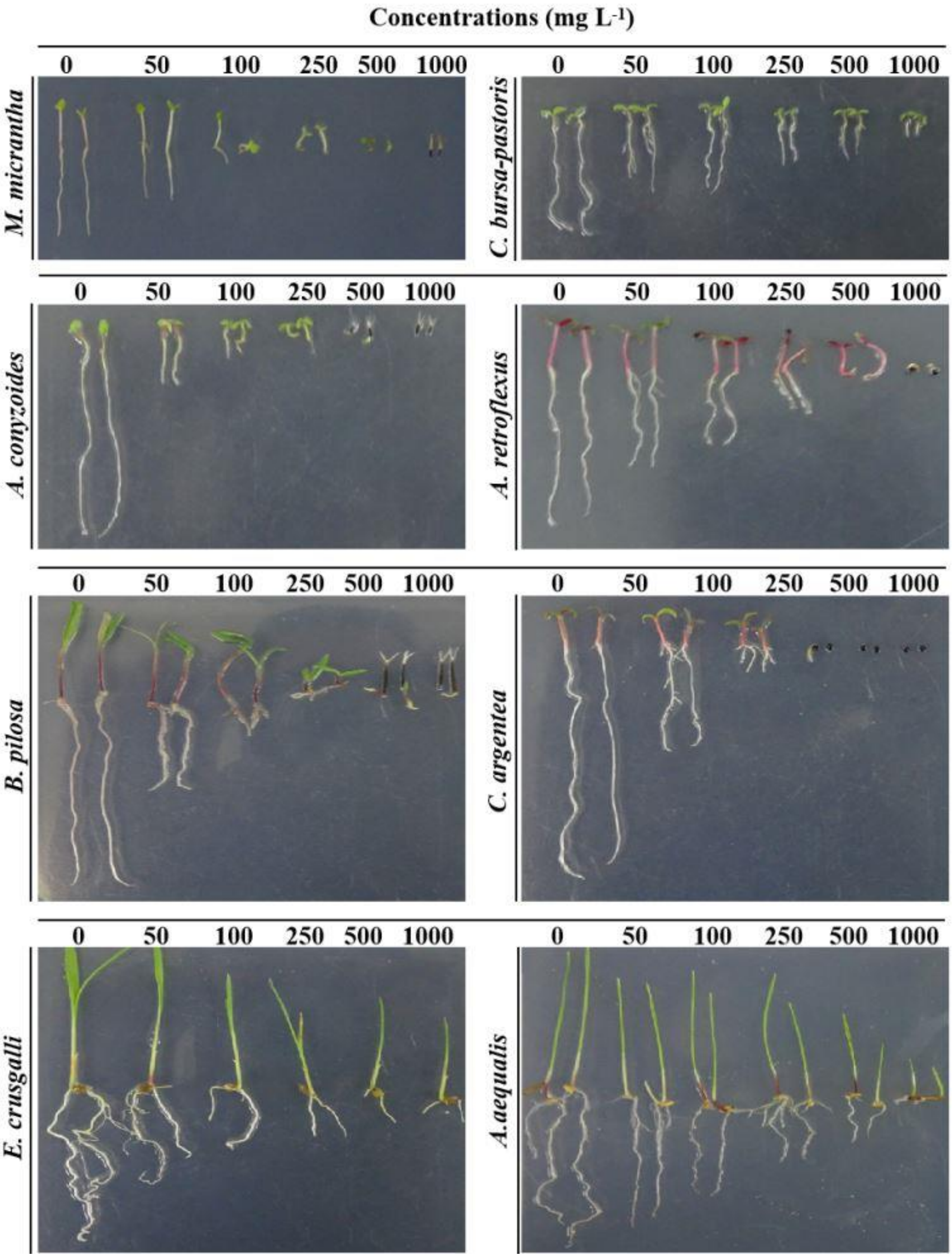

Figure S12. Inhibitory effect of dirhamnolipid against 8 tested weeds (7d).

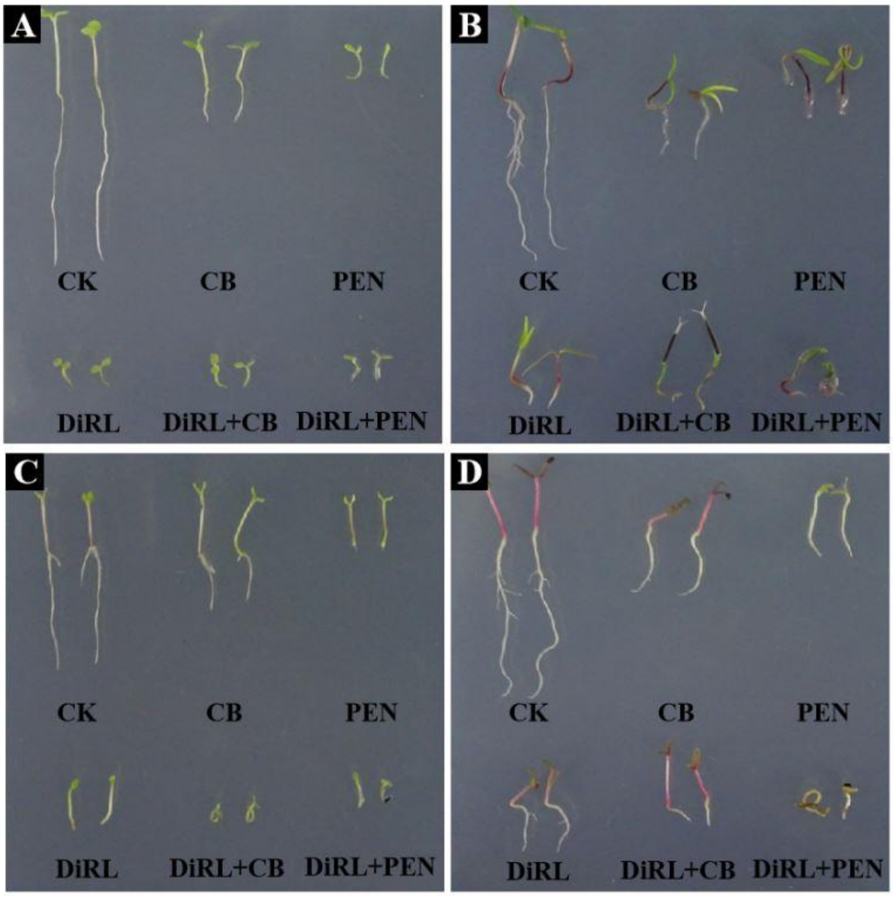

Figure S13. Synergistic effects of dirhamnolipid combined with cyhalofop-butyl or penoxsulam on the 4 tested dicot weeds at the concentration of 100 mg L^{-1} (7d). (A) *A. Conyzoides*; (B) *B. pilosa*; (C) *M. micrantha*; (D) *A. retroflexus*. CK: negative control. CB: cyhalofop-butyl. PEN: penoxsulam. DiRL: dirhamnolipid. DiRL+CB: dirhamnolipid combined with cyhalofop-butyl. DiRL+PEN: dirhamnolipid combined with penoxsulam.
